# Glatiramer Acetate in Treatment of Multiple Sclerosis: A Toolbox of Random Co-Polymers for Targeting Inflammatory Mechanisms of both the Innate and Adaptive Immune System?

**DOI:** 10.3390/ijms131114579

**Published:** 2012-11-09

**Authors:** Babak Jalilian, Halldór Bjarki Einarsson, Thomas Vorup-Jensen

**Affiliations:** Department of Biomedicine, Aarhus University, Wilhelm Meyers Allé 4, Building 1242, DK-8000, Aarhus C, Denmark; E-Mails: babak.jalilian@microbiology.au.dk (B.J.); hbe@ki.au.dk (H.B.E.)

**Keywords:** glatiramer acetate, copaxone, biosimilar, integrin, immunotherapy

## Abstract

Multiple sclerosis is a disease of the central nervous system, resulting in the demyelination of neurons, causing mild to severe symptoms. Several anti-inflammatory treatments now play a significant role in ameliorating the disease. Glatiramer acetate (GA) is a formulation of random polypeptide copolymers for the treatment of relapsing-remitting MS by limiting the frequency of attacks. While evidence suggests the influence of GA on inflammatory responses, the targeted molecular mechanisms remain poorly understood. Here, we review the multiple pharmacological modes-of-actions of glatiramer acetate in treatment of multiple sclerosis. We discuss in particular a newly discovered interaction between the leukocyte-expressed integrin α_M_β_2_ (also called Mac-1, complement receptor 3, or CD11b/CD18) and perspectives on the GA co-polymers as an influence on the function of the innate immune system.

## 1. A Brief Introduction to the Etiology and Symptoms of Multiple Sclerosis

Multiple sclerosis or MS was first described by Jean-Martin Charcot in 1868 [1]. MS is a disease of the central nervous system (CNS) causing non-traumatic chronic neurological disability, mostly in young and middle-aged adults, affecting over 2 million people worldwide [2]. The prevalence of MS may vary between different populations with incidences from 2 to 150 per 100,000. It is more common among Caucasian races in temperate regions [2,3] where MS affects 1 out of 1000 people [4]. The debut is typically occurring at the age 20–45 and women are almost twice as often affected as men [5]. There is a North-to-South gradient distribution in the MS frequency, however, with many exceptions [6].

The pathophysiology of MS has not been entirely mapped out and appears to involve multiple factors [7]. The most significant pathohistological characteristics are localized areas of demyelination in the CNS and relative preservation of axons. There are periods of focal demyelination followed by complete or partial re-myelination [8]. There is evidence that protein components of the myelin sheath stimulate the inflammatory response, which contributes to the demyelination of nerves. The process is, however, complicated and involves both lymphocytes and myeloid cells of the immune system. Experimental evidence suggests that proteins, such as myelin basic protein (MBP), act as so-called autoantigens [9], which triggers an inflammatory response towards the host tissue. The role of the immune system in MS is strongly supported by the observation that immune suppressive treatment, discussed further below, seems at least partially to be able to slow down neural decay and ameliorate symptoms. Demyelination leads to decreased conductivity or conduction block that results in neurological symptoms of MS disease. Periventricular infiltrations of lymphocytes and macrophages manifest as Dawson fingers representing demyelinating plaques through *corpus callosum* as observed by magnetic resonance imaging (MRI) modality. In addition to white substance affection, the grey matter of the CNS can also be affected [10].

**Figure 1:**
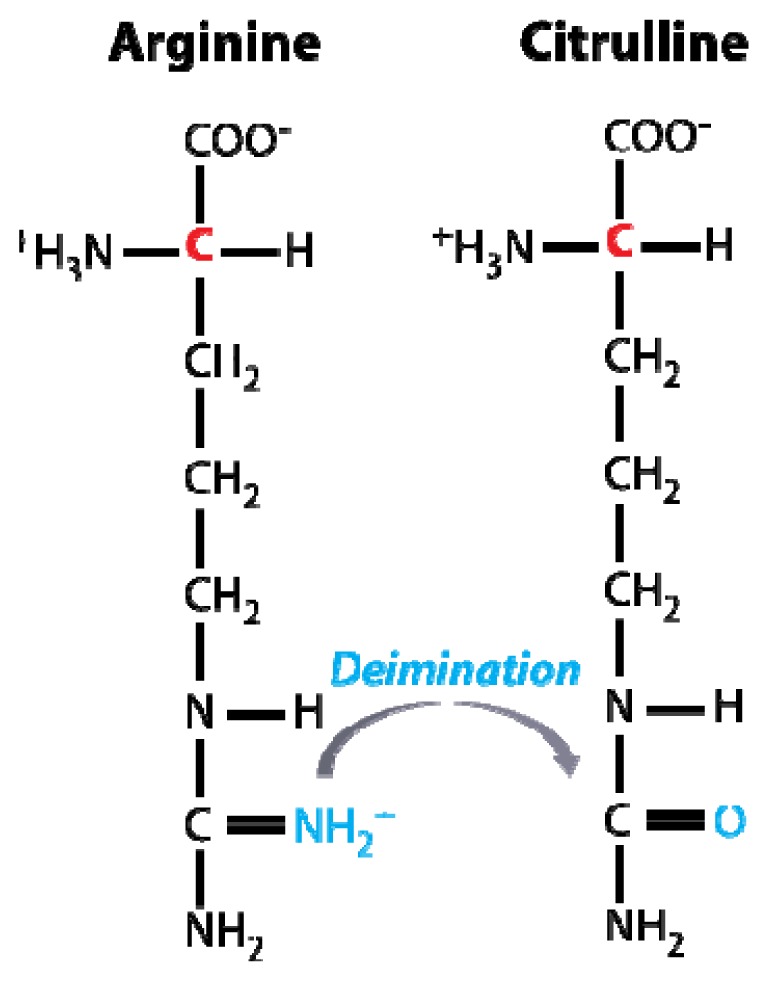
Schematic representation of the citrullination (or deimination) of the free arginine amino acid. In proteins, arginine restudies are converted into citrulline by Ca^2+^-dependent enzymes *i.e.*, peptidylarginine deiminases, of which at least six forms are known [22,23].

There is strong evidence that a combination of genetic predisposition and exposure to one or more environmental factors are linked to the etiology of MS [11–13]. The vitamin-D status, particularly in geographical regions with a limited sun light exposure, and cigarette smoking [14], have been suggested as the most consistent risk factors. Furthermore, exacerbation of MS is often associated with stress [15]. Links to infectious diseases have been suggested, both from experimental studies as well as from clinical investigations. These studies included work on bacterial antigens inducing an autoimmune response [16] as well as several studies on the role of Epstein-Barr virus (EBV) infection [17–19] and endogenous retroviruses [20]. These are potential sources of microbial manipulation of the immune system leading to excessive or uncontrolled immune responses. For the discussion in Section 5, it is of considerable interest that viral infections may alter the level of post-translational modifications of proteins expressed by infected cells, both affecting cellular gene transcription [21] and protein structure. Specifically, MBP in the human body is not a homogeneous species of molecules and present itself as a group of charge isomers [22]. This diversity in charge, results from the deimination of arginine side chains, producing a citrulline residue (Figure 1).

Since the positively-charged side chains are important in forming contact with the negatively-charged membrane lipids, as well as in certain structural properties of MBP, changes in the content of citrulline may both affect the membrane-association of MBP, inducing structural changes that expose epitopes of importance for the autoimmune recognition, and generate novel epitopes that are recognized by the immune system [24,25]. Finally, hereditary factors may play an important role in the development of MS [26] considering the statistics that approximately 15% of MS patients have a relative with MS and children of parents suffering from MS have 1–2% higher risk of developing MS compared to the MS risk factor in the same population. It is classic observation that certain major histocompatibility complex (MHC) alleles increase the risk of MS [27,28] by a factor of three as reported for the haplotype DRB1*1501 [29]. Experiments in animals also suggest MHC molecules to be a determinant in developing MS-like symptoms [30,31].

The pathogenesis of MS leads to four standardized clinical categories, namely relapsing remitting MS (RRMS), primary progressive, secondary progressive, and progressive relapsing MS [32]. Moreover, a significant portion of patients with clinically isolated syndrome (CIS) will develop a clinical definite MS [33]. The RRMS is observed in approximately 80–85% of the MS cases. This condition is clinically represented as repeated outbreaks of disease (attacks or relapsing form) with more or less complete recovery after each attack. The neurological dysfunction is seen usually to increase for each relapse with potential development of secondary progressive MS after some years. In some patients, MS is represented clinically by mild neurological symptoms with very few attacks and little or no impairment. However, on average, MS patients relapse 0.8 times per year and 15% of them enter the chronic progressive MS category (secondary progressive MS) [34,35]. The clinical findings are typically a combination of neurological deficits involving paresis, hyperreflexia, clonus, Babinski’s sign and increased tone, sensory dysfunction, ataxic eye movements, and loss of abdominal reflexes. In the case of MS, optic neuritis visual reduction, and color blindness has been reported [36,37]. Lesions in the brainstem and the cerebellum can cause manifestations such as nystagmus, dysarthria and ataxia or balance disorders as well as Lhermitte’s sign suggesting spinal involvement at a cervical level.

The consequences of having MS are often profound. It has been noted that MS disability progression follows a two-stage process [38] with approximately 10 years reduction in life expectancy [39]. The best prognosis is within the RRMS group and favorable prognostic factors are young age, sensory and visual symptoms and long intervals between acute exacerbations in the first two years after diagnosis [40]. Disease progression can stop at any given time. MS can cause diverse neurological deficits depending on the degree of dissemination and location of inflammation. The symptoms develop over minutes to days and possibly even longer, however, with the sub-acute development of symptoms being the most common. In rare cases, MS begins with acute and significant neurological deficits. Common onsets of symptoms are diplopia, unilateral optic nerve affection, paralysis, incoordination or imbalance, sensory disturbances and impaired bladder and/or bowel control. In early-stage MS the first symptoms are often weak and fast transient. The symptomatology in late stage MS varies but frequent problems are painful spasms, ataxia and dysarthria and paroxysmal symptoms like trigeminal neuralgia. More than 60% of the patients experience MS-related pain [41] and up to 50% are considerably affected by depression [42].

## 2. Differential Diagnosis of Multiple Sclerosis

The diagnosis can solely be made on the basis of medical history and clinical findings from a neurological examination when other causes are excluded. This requires medical history of neurological deficits in at least two distinct phases and the clinical findings from at least two discrete lesions in the CNS. In most cases however, the diagnosis is based on anamnesis, physical examination, results from MRI and lumbar puncture *via* detection of oligoclonal bands of immunoglobulins in the cerebrospinal fluid (CSF) [43] and/or on visually-evoked electrical potentials (VEP) recorded from the nervous system [44,45].

MRI, CSF analysis, VEP, somatosensory and motor evoked potentials can all provide important information and can be of great importance when the clinical presentation alone does not provide certainty for the diagnosis and to exclude differential diagnosis. MRI scanning of the CNS shows in typical cases multiple high signal areas in the white matter on a T2 sequence. MRI is the most sensitive method, although it does not have optimal sensitivity and specificity causing both risk of over-diagnosis and over-treatment of MS [46]. In exceptional cases, MRI findings can be negative even in clinically established MS and there are not always correlations between the imaging outcome and the clinical picture itself.

## 3. Anti-Inflammatory Treatments of MS

At present, there is no curative treatment of MS. The goal of treatment is to improve the quality of life, reducing the duration and frequency of attacks and thus potentially reduce progressive development of malfunctioning. Rehabilitory treatments are often needed due to bladder dysfunction, constipation, neurogenic pain, spasticity and psychosocial problems. However, it is arguably the case that anti-inflammatory treatments are leading in relieving the symptoms of MS. Their effectiveness also shows the importance of the immune system in developing MS.

A number of relatively simple chemical compounds exert a beneficial effect on MS, probably at least in part as a consequence of an immunosuppressive influence through inhibition of cell division. A temporary improvement is often obtained by using glucocorticoids monotherapy when other treatments are not effective or are not feasible. Typically, 3–5 days of administration of methylprednisolone intravenously, aiming to reduce the duration and number of individual relapses [47]. RRMS treatment with glucocorticoids may alternatively be given orally. Mitoxantrone is an antineoplastic drug which inhibits topoisomerase enzymes thus inhibiting RNA and DNA synthesis, and as a result is confined in highly active RRMS or secondary progressive MS with superimposed attacks [48]. Drugs like azathioprine (6-mercaptopurine) and methotrexate may reduce the relapse rate in MS patients, but are used infrequently due to sparse evidence of improvement [49–52]. A perhaps surprising source of anti-inflammatory treatment is derived from the use of statins. The best-described pharmacological effect of treatment with statins relates to their function as plasma cholesterol-lowering agents through the activity as a 3-hydroxy-3-methyl-glutaryl-CoA reductase. However, statins such as simvastatin and lovastatin also acts as allosteric inhibitors of integrin α_L_β_2_ (also named lymphocyte function-associated antigen-1 or CD11a/CD18) ligand binding [53,54]. This has been documented through studies on the function of leukocytes *in vitro*, structural studies on the α_L_ ligand binding domain in complex with lovastatin, and the observation that statins apparently exert an immunosuppressive effect in treated patients [53–55]. Several studies have investigated simvastatin and atorvastatin for effects on MS. The findings have so far remained negative, signifying no improvement in condition of treated MS patients [26,56]. It is interesting however, that lovastatin to our knowledge has not been tested in these trials. Furthermore, according to experiments *in vitro*, statins such as simvastatin and lovastatin may not inhibit the function of α_M_β_2_ suggesting that therapy with, e.g., simvastatin, would not block the function of all relevant cell adhesion molecules possibly explaining the lack of efficacy.

Direct interference with the cellular constituents of the immune system in patients can be obtained by plasmapheresis as adjunctive therapy, which is used in some cases of primary progressive MS and often considered as adjunctive therapy of exacerbations in relapsing forms of MS [57].

The pharmacological agents mentioned above are broad-acting drugs with several influences that potentially are important for reducing MS disease progression or severity. However, there are several significant side effects of these treatments. Biological therapy—typically with purified or recombinant human proteins, monoclonal antibodies, or receptor analogues—far more specifically targets certain functions of the immune response. Human immunoglobulin may be an alternative for patients with RRMS and when other treatments are not feasible or effective. The documentation is limited, but one study has recently shown some effect at one year of observation [58]. While the pharmacological mode of action is not clear, evidence from other inflammatory diseases of the CNS, seems to suggest that at least partial saturation of cellularly-expressed Fc receptors may play a role [59]. Interferon beta is indicated in RRMS and it appears to slow disease progression in selected patients [60]. Early treatment with Interferon beta, *i.e.*, after the first attack, may extend the time of conversion to clinically definite MS [61,62]. Monoclonal antibodies to cell adhesion molecules have proven remarkably effective in treating disorders involving excessive inflammation. Yednock *et al.* showed [63] that a function-blocking antibody to the α_4_ chain of the integrin α_4_β_1_ (also named very-late antigen-4 or CD49d/CD29) and α_4_β_7_ in rats, prevented experimental autoimmune encephalomyelitis (EAE), which is a well-established albeit not unproblematic animal model of MS [64]. A fully humanized antibody (natalizumab or Tysabri^®^) is indicated as a monotherapy in very active RRMS stage, despite treatment with interferon beta. It has been shown that the treatment reduces relapsing rate at 1 year from 0.75 to 0.25 (68% reduction) and the number of new or enlarged brain lesions on MRI reduces by 83% [65,66]. Development of neutralizing antibodies to Natalizumab may however lead to a reduced treatment effect and should be controlled during treatment [67,68]. In addition, it should be noted that progressive multifocal leukoencephalopathy is associated with Natalizumab treatment [69]. These side effects were also observed for function blocking antibodies to other cell adhesion molecules such as integrin α_L_β_2_[70]. As reviewed elsewhere [71], a future perspective may involve the use of various nanomedicine formulations of integrin ligand binding competitors as a safer alternative to function-blocking monoclonal antibodies. However, the presence of soluble adhesion molecules in human plasma, generated by proteolytic shedding, adds complexity to regulating the outcome of blocking receptor function with either monoclonal antibodies or other means [71]. Several strategies now also target B-lymphocytes. The CNS of MS patients is both the target of the immunopathological process as well as a site of local antibody production. B cells can increase or dampen CNS inflammation. Since B-cell depletion is a promising therapeutic strategy their proinflammatory effects seem to be more prominent in most patients [72].

Copaxone^®^ is an immunomodulator with glatiramer acetate as active ingredient. In 1987, Bronstein *et al.*, studied and compared a group of MS patients receiving GA against a placebo group in which, the group receiving GA, showed 30% more improvement in their disability score [73]. Since the approval of Copaxone^®^ for MS treatment in the US in 1996, it has remained popular for treatment of MS considering the life-threatening side effects of other competitors such as Tysabri and Mitoxantrone [74]. In two different projects Miller *et al.* studied the long term (up to 22 years) effects of Copaxone^®^ as the sole therapy for relapsing-remitting MS (RR-MS) patients. In these studies, the long term use of Copaxone^®^ did not influence the efficacy and safety showing its important features as a safe and reliable treatment for MS considering the chronic nature of the disease [74–76]. The superiority of GA treatment over placebo has been indicated by a double-blind, randomized, placebo-controlled study of the effects of GA on MRI. This study shows, by its highly statistical significance, that the total number of CNS lesions on T1- and T2 weighted MRI, involving 239 patients with RRMS, was reduced (*p* = 0.003), showing in addition a significant reduction by 33% in relapse rate [77]. A multicenter double-blind placebo-controlled study from 1995 then demonstrated GA to reduce the number of new relapses by approximately 30% without significant side effects [78] leading to regulatory authorization of MS treatment from 1996 in the US and 2000 in Europe. In addition, a meta-analysis from Boneschi *et al.* supported these findings by bringing to light a highly statistical significant difference between the intervention groups receiving GA and the placebo group as to the following endpoints; adjusted annualized relapse rate, adjusted risk ratio for the on-trial total number of relapses and time to first relapse [79]. The research group stated that the meta-analysis reaffirms the effectiveness of GA in reducing relapse rate and disability accumulation in RRMS, at a magnitude comparable to that of other available immune modulating treatments. They also suggested that the efficacy of GA is not significantly influenced by the patients’ clinical characteristics at the time of treatment initiation. However, a recent meta-analysis by La Mantia *et al.* states otherwise [80]. All randomized controlled trials comparing GA and placebo in patients with definite MS, whatever the administration schedule and disease course, were eligible for their review. The objectives, in their study, were to verify the clinical efficiency of GA in the treatment of MS patients with RRMS and progressive-course MS (PMS). Five hundred and forty RRMS patients and 1049 PMS were selected for the analysis and they concluded that GA has a partial efficiency in RRMS, *i.e.*, in terms of relapse-related clinical outcomes, without any significant effect on clinical progression of disease measured as sustained disability. The drug showed no effect in progressive MS patients and, therefore, continuing the treatment with GA seems to have few beneficial effects in RRMS, and no significant impact in PMS patients [80]. In other words, the study showed no beneficial effects on disease progression in two MS forms. However, they found a slight reduction in the frequency of relapses in RRMS patients and as mentioned no positive improvements in the conditions of the PMS group were observed. Side effects as flushing, chest tightness, sweating, palpitations, anxiety and local injection-site reactions occurred quiet frequently, but no major side effects were observed. Currently it is recommended that the treatment is terminated or switched to another approved treatment, if the frequency or severity of clinical relapses become worse during the GA-treatment or that the progression of disease is to such an extent that the patient no longer is thought to benefit from the treatment (*i.e.*, GA is not indicated for use in a progressive state of the disease). Patients receiving treatment with GA should be informed on side effects according to *lege artis*, health and medicines authorities, and that a reaction with one or more of the following symptoms may occur within minutes after injection: mild injection-site reaction, manifested by erythema, inflammation, and induration. The most remarkable side effect has been reported to be systemic post-injection reaction that occurred in 10% of patients manifested by flushing, chest tightness, palpitations, dyspnea, and anxiety [81]. Most of these symptoms are transient and disappear spontaneously and without sequelae. There is no risk that special populations are at a particular risk for these reactions. Nevertheless, caution should be applied when GA is administrated to patients with cardiac disease and plasma for its content of GA, should be monitored during and after treatment. Serious hypersensitivity reactions (e.g., bronchospasm, anaphylaxis or urticaria) may rarely occur [82]. There are no adequate data for treatment during pregnancy and breastfeeding (*i.e.*, no information can be found on GA excretion, GAs’ metabolites or its antibodies in breast milk). Animal studies are insufficient with respect to pregnancy, on embryonic development, delivery and postnatal health. Therefore treatment with GA is not recommended during pregnancy [82]. Renal function should be monitored regularly in patients with renal impairment, but there is no evidence of glomerular deposition of immune complexes in humans. However, the possibility cannot be excluded as GA-reactive antibodies have been found in patient receiving GA treatment [83,84]. The peak in antibody titers to GA was here reached on average after 3–4 months of treatment, followed by a reduction in the titers. In conclusion, the current scheme of GA-treatment with 20 mg injection subcutaneously per 24 h is considered a good option for RRMS patients when walking mobility is persevered and there are clinical signs of disease activity, *i.e.*, evidence of relapses during previous two years. In addition, GA-treatment is indicated in clinically well-defined first demyelinating episode and when alternative or differential diagnosis has been excluded. This is not trivial and it must be noted that GA prescription and administration should only be done by a physicians experienced in neurology and in treating MS patients. Furthermore, the treatment should be followed up with clinical examination three and six months after treatment and followed up every six months, with registration of number of attacks, side effects and objective neurological examination. Currently, it is not known how long patients should be treated.

While the clinical data supports a beneficial effect of GA in the treatment of MS, there is considerable uncertainty as to what are the major pharmacological modes of action (PMA). As presented in the following sections the chemical nature of the compound is complex and for this reason sometimes misrepresented unintentionally in the scientific literature.

## 4. What Is Glatiramer Acetate?

As a starting point, it is beneficial to recapitulate briefly the historical development of GA, as it can be extracted from the original reports and a few reviews detailing the chemistry [85–87]. In the mid-1900, the science of amino acid polymerization had moved to a level where it seemed possible to make synthetic high-molecular weight compounds as models of biopolymers, here, of course, most notably proteins. Through the work of Katchalski-Katzir *et al.*, it became possible to make poly amino acids from their *N*-carboxy-α-amino acid anhydrides (NCA), in some cases modified with appropriate protecting groups, which were removed to generate the final product in processes also introduced from this work [88,89]. Initially, homopolymers such as poly-l-lysine and poly-l-glutamate were made [90]. Even in some of the first experiments average lengths of the polypeptides reached more than 30 units and procedures for making polymers with a degree-of-polymerization (DP) at approximately 200 was soon reached [91]. The DP is influenced by temperature, with low temperatures favoring the formation of polymers with high DP when the reaction takes place in solution. In inert media the reaction requires initiation by addition of amines or strong bases such as NaOH. Interestingly, the choice of initiator, e.g., primary, secondary or tertiary amines, and molar fraction of the initiator may also influence DP of the polymers. With such control over the chemistry of polymers made according to this scheme, the polypeptides turned out to be instrumental in forming a solid basis for our current understanding of protein structure. Among many significant achievements, the alpha helical structure in proteins predicted by Pauling and Corey [92] was found by Perutz using poly-γ-benzyl-l-glutamate [93]. Also, the ability to make polymers as model of collagens, using block polymers of poly (Pro-Gly-Pro), identified the critical chemistry behind the triple helical structure attributed to collagen fibers by Rich and Crick [94] and was the first example of synthetic polymers forming a trimer. This principle was used subsequently in numerous investigations on the properties of collagen [95]. In addition to X-ray diffraction on the fibrous material, the use of ultraviolet rotatory dispersion spectroscopy was quickly introduced to confirm the formation of secondary structure in the polymers in various aqueous environments [91].

**Figure 2:**
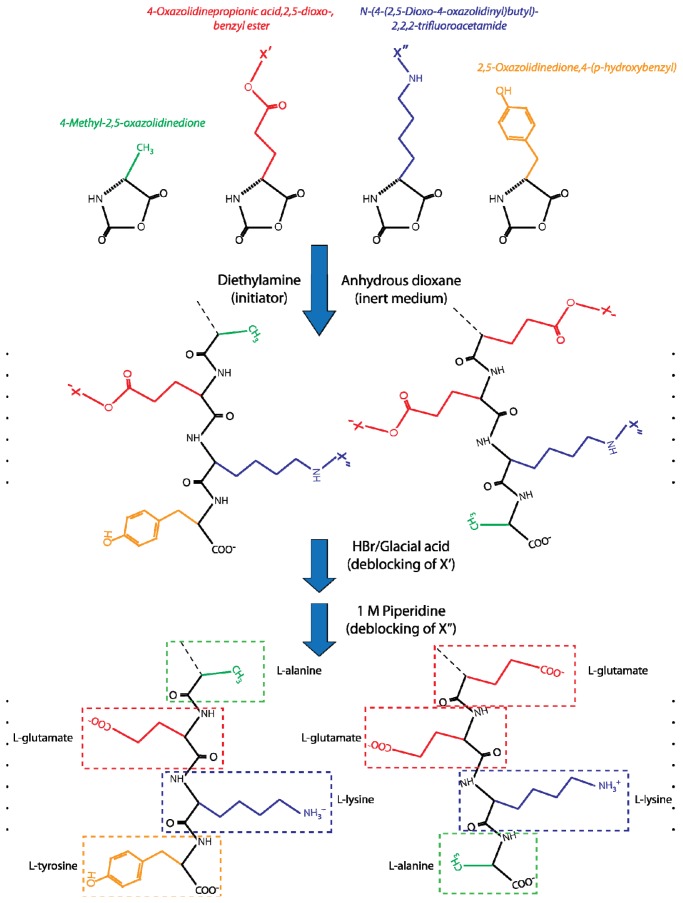
Schematic representation of some of the reactions forming Cop-1 co-polymers [91,104]. The co-polymers are made from the NCA of l-alanine (4-methyl-2,5-oxazolidinedione), and l-tyrosine (2,5-oxazolidinedione,4-(p-hydroxybenzyl)). l-glutamate is included with a protecting group (X′: γ-benzyl ester) as 4-oxazolidinepropionic acid, 2,5-dioxo-, benzyl ester. l-lysine is included with a protecting group (X″: ɛ,*N*-trifluoroacetamide) as *N*-(4-(2,5-dioxo-4-oxazolidinyl) butyl)-2,2,2-trifluoroacetamide. As examples, the C-termini of two co-polymers are shown in the panel. Following the polymerization, which may take place at room temperature [103], X′ was removed by treatment with hydrogen bromide in glacial acid and X″ was removed by treatment with 1 M piperidine, which produces the final acetate salt of co-polymers of the four amino acids.

The chemistry of GA is tightly linked with the observation that the polypeptide polymers have various biological activities, including the ability to act as a substrate for enzymes [96] as well as being antigenic, *i.e.*, able to stimulate an immune response as shown in several reports by Sela and Arnon [97–100]. A classic experimental animal model for the development of MS in humans involves injection of MBP and adjuvant (typically “Complete Freund’s adjuvant”, which contains strongly immunostimulatory components) into animals, mostly rodents but also primates [64]. As suggested by the name, this protein is a highly positively charged constituent of the myelin sheath surrounding the nerve axon [22]. In consequence of MBP’s antigenic properties, this injection directs an immune response to nerve tissue causing encephalitis with some similarities to the pathology of human MS. Since MBP obviously is a host protein, this model has greatly served to strengthen the idea that MS is an autoimmune disease and the model is usually referred to as EAE. Previously it had been observed, however, that MBP can suppress EAE when injected in the absence of adjuvant [87,101]. These capacities prompted Teitelbaum *et al.* to synthesize a polypeptide from the acid anhydrides of γ-benzyl-l-glutamate, ɛ,*N*-trifluoroacetyl-l-lysine, l-alanine, and l-tyrosine with molar ratios of 1.9:4.6:6.0:1.0 in the formed polymers [102]. This formulation was named Copolymer-1 or Cop-1. In this way, the four cardinal biochemical properties of amino acid side chains, *i.e.*, side chains with negative or positive charge or polar or hydrophobic groups, were represented when the protecting groups were removed from the glutamate and lysine residues. Compared with the scientific publication [102] further detail on the synthesis methodology can be found in patent filed in 1971, which identified diethylamine as initiator of the polymerization carried out at ambient temperature in anhydrous dioxane [103]. This removal was carried out by de-blocking of the γ-benzyl-l-glutamate affected with hydrogen bromide in glacial acid, followed by removal of the ɛ,*N*-trifluoroacetyl from lysine residues by 1 M piperidine [103] (Figure 2).

In the case of Copolymer-1, the ratios of NCA were chosen to mimic the stoichiometry of side chains in MBP with negative or positive charge or polar or hydrophobic groups, which at the time was essentially the only known property of MBP’s structure. By making random co-polymers with this stoichiometric composition at the very least some of the immunoregulatory properties of MBP would be presented by synthetic polymers. Indeed, it was found that Cop-1 worked to block the encephalitis [102]. By contrast, other polymers, including one similar to Cop-1 except not containing lysine, could not suppress EAE [102]. It should be noted that the co-polymers forming a part of the currently used GA formulation apparently are synthesized in a way which results in ratios of alanine, lysine, glutamate, and tyrosine in the GA polymers of 1.4:3.4:4.2:1, which is different from the original report on Copolymer-1. To our knowledge, no explanation for this difference has been indicated in the scientific literature. For the discussion below, it is also of interest, that the patent mentioned two other polymers consisting of tyrosine, aspartate, alanine, and lysine or glutamate, alanine, and lysine, respectively, leading to similar results as the Cop-1 formulation for treatment of EAE [103]. These polymers all had a relatively high content of lysine and hence a net positive charge, which, as noted by Teitelbaum *et al.*, pointed out the importance of this amino acid and the positive charge to trigger suppression of EAE by the polymers [102]. However, the chemistry of these co-polymers was different from Cop-1 and hence not matching MBP in the way the Cop-1 does. Indeed, co-polymers chemically different from Cop-1 may exert a stronger suppressive influence on EAE than Cop-1 [105].

By definition, co-polymers such as those found in GA are derived from two or more monomers, *i.e.*, in the case of GA the four acid anhydrides mentioned above, unlike homopolymers which are derived from only one type of monomeric species. In the medical literature GA is, however, rarely indicated as containing co-polymers, probably, one may speculate, because pharmacological treatment with even simpler polymeric substances is still rather limited. Indeed, GA was the first synthetic polymeric pharmacological agent to be used in internal medicine. In this situation references to GA as belonging to the class of co-polymers are rarely added in databases indexing drugs, although an expert probably would be able to infer from the provided information that GA is likely to be a co-polymer. The exact nomenclature may seem only of semantic interest, however. By contrast, a more important challenge in understanding the nature of the drug resides in the extraordinary structural variation found amongst the synthesized co-polymers. As a typical result of polymer synthesis, the copolymers are not size uniform. Indeed, the clinically-used formulation of GA contains copolymers with a range of *M*_r_s ranging from 5000 to approximately 9000 corresponding to DPs of 45–80 residues. As noted by Arnon and Sela [87], this is nevertheless a narrow distribution in size compared to most products of polymer synthesis, which is a result of the chemistry of the polymerization process, where the polymers grow only from coupling with the acid anhydride monomers and not through coupling with polymerized species [87,91]. Another limitation on the size heterogeneity comes from the observation that termination of the polymerization occurs mainly through an intermolecular reaction with acid anhydrides creating a terminal carboxyl group [87,91]. A striking contribution to the heterogeneity among copolymers comes from the randomness of the sequences. If the co-polymers contain approximately 50 residues, the number of possible combinations with four polymerizing amino acids equals ~4^50^ (*i.e.*, equal to ~10^30^) possible peptides. Since patients receive in the order of ~10^17^ such polymers per injection, a patient will never in his or her lifetime receive chemically identical co-polymers. Furthermore, this variation among the co-polymers also affects the composition of the purified material where a batch-to-batch variation make the molar fraction of l-glutamate, l-alanin, l-tyrosine, and l-lysine vary in intervals of 0.129–0.153, 0.392–0.462, 0.086–0.100 and 0.300–0.374, respectively.

The chemical heterogeneity makes GA hard to characterize by available methodologies. Interestingly, this situation is remarkably similar to another polymeric substance, which is used in the clinic, namely heparin. Heparin is a random co-polymer with an average DP of 20, polymerized from a choice of 32 disaccharide units [106]. This produces a theoretical diversity of ~10^14^ distinct co-polymers—if not as many as for GA, then still a considerable heterogeneity for a compound. An important market regulator of the costs of pharmacological treatment, and hence overall cost of health care systems, is the availability of generic drugs. Usually, a generic drug would be pharmaceutically equivalent to the original formulation, meaning that it contains the same active ingredient. However, in the case of enoxaparin sodium injection (Lovenox^®^), an anticoagulant with biologically-derived heparin as the active ingredient, the manufacture of a generic product caused significant challenges in the absence of precise chemical insight on the structure of the heparin co-polymers. However, as judged from available accounts in the literature and on the internet the company Momenta Pharmaceuticals (Cambridge, MA, USA) applied advanced methodologies to sequence heparin co-polymers originally developed at the Massachusetts Institute of Technology, by use of matrixassisted laser desorption ionization mass spectrometry, hereby obtaining information sufficient for bringing generic Lovenox on the market for prevention of deep vein thrombosis [107]. Such a product is now referred to as a “biosimilar”, or “follow-on biologic”, indicating that the pharmacological innovative aspect was made with the prior manufacture of another drug [108]. Apparently, Momenta Pharmaceuticals is currently in the pursuit of making generic Copaxone^®^, here named M356, which is likely also to involve technologies capable of characterizing complex protein drugs albeit the specifics are not clear. A second company, Synthon (Nijmegen, The Netherlands) is also pursuing the manufacture of generic Copaxone according to their web site, but details on the process of characterization or manufacture is also in this case not available to our knowledge. Finally, both in the case of heparin-based anticoagulants and GA, products with a more well-defined chemical nature were made. Following the “heparin crisis” where naturally-derived heparin contaminated with chondroitin sulfate caused adverse clinical events [109,110] considerable efforts have been invested in making synthetic heparin [109,111]. Similarly, efforts have been invested in making peptidic substances with a considerable less heterogeneous composition than Cop-1 [112]. While it is unclear if such a strategy is currently being commercially explored in the case of GA, at least in principle it suggests one route of making competing drugs.

## 5. The Pharmacological Modes-of-Action of GA

As pointed out above, the extraordinary diversity of the GA co-polymers raises an important question as to how the effects in the EAE model and the human MS disease are obtained. As identified below, the suggested mechanisms can largely be divided into three groups, two of which relate to an influence on the inflammatory response. Here, focus is placed on the possible influences of GA on the adaptive and innate immune system. Finally, a brief mentioning is made of a neuroprotective effects, that, however, also appears to involve cells of the immune system.

The adaptive immune response critically relies on the interconnected function of T and B lymphocytes with the T cells being an important source of regulatory cytokines and B lymphocytes a precursor state of the antibody-producing plasma cells. A considerable body of studies has addressed the influence of Cop-1 or GA on these cells, mainly through the use of the EAE model as well as from observations made on clinical samples. Some of these studies are reviewed below. A hallmark of adaptive immunity is sequence-specific recognition of peptidic motifs by the T and B cell receptors, which are formed through somatic recombination and mutation of germ line genes and makes a large diversity of receptors permitting the recognition of large number of antigens. Clonal selection in lymphoid organs both removes autoreactive clones and selects those clones with receptors strongly recognizing antigens.

**Figure 3:**
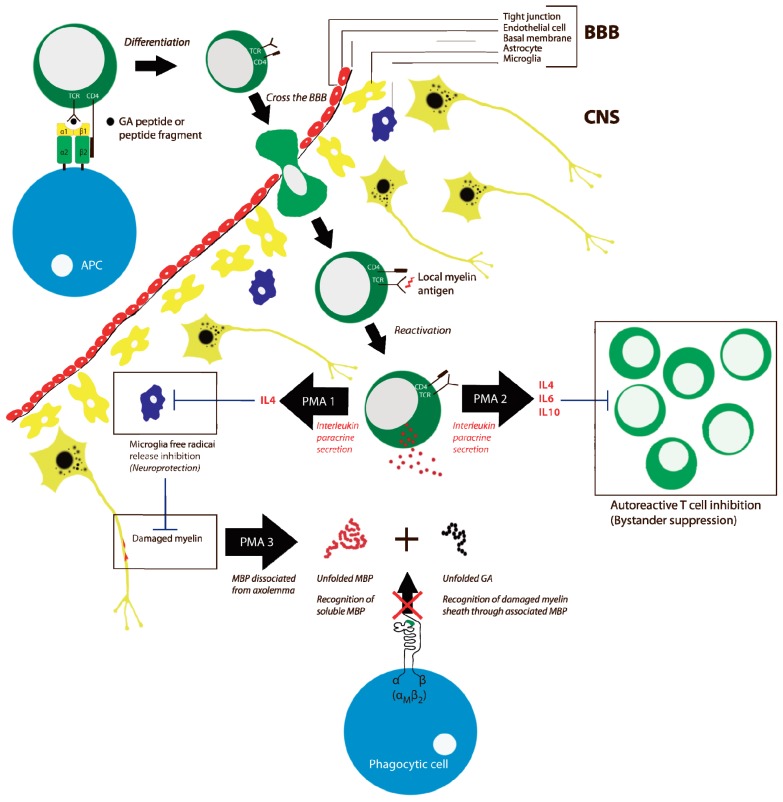
Some of the pharmacological modes-of-action (PMA) of GA-treatment of MS patients. Three selected mechanisms are indicated, representing neuroprotective effects through (1) IL-4 mediated suppression of microgial free radical release [128,129] or modulation of the adaptive immune response through (2) by-stander suppression of autoreactive T cell proliferation [122,127]. Based on experiments *in vitro*, Stapulionis *et al.* suggested an influence of GA on the innate immune system through (3) inhibition of phagocytic damage to the myelin sheath by inhibition of integrin α_M_β_2_ binding to the exposed MBP [130].

Following the initial study on Cop-1 suppressing EAE [102], it was demonstrated that Cop-1 induced the formation of specific antibodies to the co-polymers, which cross-reacted with MBP [113]. A correlation between this immunological cross-reactivity and the suppression of EAE clearly pointed that injection of the co-polymers manipulated the immune response. As noted above, another important observation was that Cop-1 may compete with MBP for binding to MHC class II molecules [114,115]. EAE may be induced by other agents than MBP, such as peptides from proteolipid protein (PLP) or myelin oligodendrocyte glycoprotein (MOG). Both of these proteins are not structurally related to MBP but carry a net positive charge (*i.e.*, with isoelectric points of ~8) at a more moderate level than MBP (with an isolectric point of ~10). Interestingly, Cop-1 also suppressed EAE induced by these agents [116,117]. It is unclear if any critical similarity exists between PLP, MOG, and MBP, but it is, of course, striking that the positive charge of these proteins and Cop-1 may be an important shared characteristic also adding to findings mentioned above that co-polymers with lysine—but otherwise no obvious mimicry of MBP—were suppressing EAE. Taken together, it is debatable if the antigenetic similarities between Cop-1 and MBP are critical for the EAE suppression. Rather, it would seem likely that Cop-1 and GA works through a more general mechanism with regard to influencing the adaptive immune system. Support for the latter point may be derived from the observation that Cop-1 also works to block the inflammatory response in animal models of cerebral stroke [118] and graft-versus-host disease [119]. In particular the study on graft-versus-host disease [119] suggests that Cop-1, and hence GA, may act as a general immunomodulatory compound not requiring a role of MBP in the inflammatory response. This is supported by an important study on GA co-polymers, *i.e.*, the clinically-used formulation, as antigen, carried out by Duda *et al.*[120]. This paper showed that the co-polymers induced CD4^+^ T cell proliferation of both naïve and memory T cells. In this sense, the GA co-polymers may act as universal antigens, which could be thought to alter T cell receptor repertoire in treated patients. However, a recent analysis failed to find such alterations in MS patients treated with GA compared to patients not receiving this treatment albeit the numbers of analyzed patients were low [121]. Another important influence of GA on T cell function was reported by Aharoni *et al.* reporting that GA generate bystander suppressor T cells and, more recently, a similar suggestion was made by Kala *et al.* pointing to GA modulation of regulatory B cells [122,123]. These cellular effects induce the synthesis of a plethora of cytokines, presumably mostly anti-inflammatory, supporting the view that several independent mechanisms contribute to the therapeutic effect of GA [124]. GA-reacting T helper cells are found in all patients with MS. During treatment with GA, these cells will differentiate in the direction of Th2 regulatory T cells, which have anti-inflammatory properties through their secretion of cytokines [120]. In the case of human MS it has been hypothesized that these cells pass the blood-brain barrier (BBB) (Figure 3) [125]. In the case of EAE Th2 cells were demonstrated to localize in the CNS [122] where myelin antigens are presented [126]. Here, they then reactivate, resulting in secretion of anti-inflammatory cytokines. These cytokines (e.g., IL4, IL-6, IL-10) inhibit the activity of potential surrounding autoaggressive T cells. This action is called bystander suppression (Figure 3) [122,127]. Despite these findings, experiments with IL-4- and IL-10-deficient mice show that GA still had beneficial effects in suppressing EAE in the absence of these two prominent Th2 cytokines. Thus, Jee *et al.* suggested that alternative modes-of-action to bystander suppression may be responsible for these effects of GA [128].

The innate immune system functions, in part, through receptors and soluble molecules encoded by germ line genes. These are evolutionary selected to encode proteins that permit the recognition of certain microbes, usually through the exposure of pathogen-associated molecular patterns that distinguishes these microbes from host cells. However, an increasing body of literature now shows that the innate immune system also plays a role in recognizing damaged tissue or molecular species of the host [131]. Such functions are at least partially mediated by cell membrane-expressed receptors that recognize structurally or decayed molecules [132].

The β_2_ integrins are exclusively expressed on leukocytes where they support cellular adhesion to ICAMs, fragments of complement component C3 deposited on target surfaces through the activation of the complement system, or protein components of the extracellular matrix such as fibrin [133,134]. The receptors are linked with the cytoskeleton through transmembrane domains and this contact, in turn, permits allosteric regulation of ligand binding through conformational changes in the receptor ecto-domain [135]. Recently, it has become clear that the full functionality of these receptors in supporting cellular migration probably requires several proteases that shed the ecto domain of β_2_ enabling the de-adhesion of migrating cells [71,136,137]. Inspired by the many findings suggesting that adhesion molecules are important drug targets in anti-inflammatory therapy, Stapulionis *et al.* investigated if GA influences the function of β_2_ integrins [130]. α_M_β_2_ (also known as Mac-1, complement receptor 3, or CD11b/CD18) and α_X_β_2_ (p150,95, complement receptor 4, or CD11c/CD18) are mainly expressed on NK cells and myeloid-derived leukocytes, such as neutrophil granulocytes and monocytes/macrophages, which are usually thought to constitute the cellular arm of the innate immune system. However, these receptors are also expressed on dendritic cells, which play a significant role in establishing an antigen-specific response by T lymphocytes. While integrin α_L_β_2_ is relatively specific in its interaction with the ICAMs, integrins α_M_β_2_ and α_X_β_2_ are far more promiscuous in their interactions with ligands. Consequently, a large number of biomacromolecules, not only including proteins and several peptides but also carbohydrates and components of the microbial cell wall were reported as ligands [71]. A chemical rationale for the underlying principles in ligand recognition by these integrins was proposed by Vorup-Jensen *et al.*[138]. According to these studies, central properties of ligands are the presence of acidic groups to coordinate a Mg^2+^ ion in the ligand binding domain and a low abundance of secondary structure. Such a system would enable phagocytes expressing these integrins to clear decayed molecular species, which are produced through the activity of, in particular, proteases, which destroys the structure of proteins. In the context of the discussion below on the binding between integrin α_M_β_2_ and GA, it should be noted that another polymer reported to bind strongly to integrin α_X_β_2_, and less strongly by integrin α_M_β_2_, is heparin [139]. As discussed in Section 4, heparin is a random co-polymer with little, if any, secondary structure in solution [106]. In this way, the observation that heparin binds α_X_β_2_ and α_M_β_2_ supports the idea that random co-polymers can be ligands for these integrins albeit the structural biology of these interactions is unclear. The ability of integrin α_X_β_2_ to preferentially bind certain unfolded proteins is shared by integrin α_M_β_2_, however, the binding by this integrin is not strengthened by a polyanionic character of the ligand as was found for α_X_β_2_[71,138,140]. Indeed, the observation that MBP is a ligand for this integrin [130] may suggest that positively-charged species bind well to the integrin α_M_β_2_. Likewise, studies using synchrotron radiation circular dichroism spectroscopy revealed that the GA co-polymers present little secondary structure, probably in consequence of the high content of lysine, which contributes a hydrophilic and positively charged character to the peptide [130]. In this sense, the GA co-polymers and MBP share biophysical characteristics since MBP in aqueous environment are a classic and well-studied examples of an intrinsically unordered protein [22]. In experiments carried out *in vitro* Stapulionis *et al.* reported that the GA co-polymers bind integrin α_M_β_2_ to a level where they may act as competitive ligand binding antagonist, blocking the binding of this integrin to MBP [130].

These *in vitro* findings inspired a suggestion on the PMA of GA. Following injury to myelin sheath of the nerve, MBP is brought into contact with the aqueous environments of the CSF, which unfolds the protein making it a ligand for integrin α_M_β_2_. It has been shown that MBP can be detected in CSF of MS patients, presumably as a consequence of demyelination in the white matter of the brain [141,142]. The interaction with integrin α_M_β_2_ promotes the phagocytic uptake of MBP, possibly in a format where MBP is part of myelin sheath fragments, which was shown earlier to be internalized by phagocytes [143,144]. Such internalization may lead to cytokine production by the phagocytes as well as a direct attack by these cells on the myelin sheath, which could affect both myelination and remyelinination processes. Studies in mice clearly suggest that there is complex interplay between leukocyte subsets in the process of healing CNS lesions [145]. Both under normophysiological conditions as well as in the pathophysiology of MS, several types of integrin α_M_β_2_-expressing cells can be found in the CNS. In particular, these include microglial cells as well as other cells of myeloid origin invading the CNS from the blood, most notably monocytes and neutrophil granulocytes under extreme circumstances [146].

For GA to be efficient in blocking the binding between α_M_β_2_ and MBP in the CNS, an obvious prerequisite is the ability of GA to pass the BBB. As a first step on the route to reach the CNS, the survival following the injection would seem particularly important. *In vitro* data and data from clinical trials in healthy volunteers indicate that after subcutaneous administration, GA is rapidly absorbed. The pharmacokinetics of GA have indeed been evaluated in mice, rats and monkeys by radiolabeling technique [147]. Maximum serum radioactivity is observed after 2 to 4 hours in monkeys. The active substance is rapidly absorbed after subcutaneous injection (20 mg daily) with 10% remaining at the injection site after 1 h [148]. There is a rapid hydrolysis to amino acids and shorter peptides and a large part of the degradation already occur in the subcutaneous tissue. Furthermore, no systemic plasma, urinary or fecal excretion of GA is indicated [148]. However, this may not impair the influence on integrin α_M_β_2_ since it is well established that even small peptides may act as binding antagonists *in vivo*. With regard to the actual site of action for the MBP binding-antagonistic effects of GA, both the report by Stapulionis *et al.*[130] as well in Figure 3, it is suggested that this would be relevant in the immediate contact between the damaged myelin sheath and an integrin α_M_β_2_-expressing phagocyte such as microglial cells or monocytes having crossed the BBB [149,150]. An important caveat to this suggestion concerns the biodistribution of GA, or more specifically, if the GA polymers are able to cross the BBB. We are not aware of any studies addressing if GA may actually reach the CNS, but an important reason to suggest such a possibility may be the dysfunction, or breakdown, of the BBB, which is thought to occur in MS. It is a classic finding that the concentration of high-molecular weight proteins such as fibrinogen increases in the CSF in the event of a break-down of the BBB and even in healthy individuals there appears to be an exchange of substances with a *M*_r_ of less than 160,000 [151]. Any proteolytic break-down products of the GA polymers, and maybe even the intact co-polymers themselves, would seem ideally suited for crossing the BBB. Furthermore, in addition to the co-polymers the GA formulation contains 40 mg/mL mannitol. The addition of mannitol is a method to bring drugs across the BBB [152]. It should be noted, however, that this addition was apparently not part of the Cop-1 formulations used in the many studies on EAE mentioned above.

It is also possible, however, that the α_M_β_2_ ligand binding antagonistic properties of GA may influence the immune response to myelin sheath by acting on leukocytes outside the CNS, *i.e.*, in plasma or secondary lymphoid tissues. A recent study by Toker *et al.* pointed that fluorescently tagged GA co-polymers are selectively bound by CD11b^+^ (*i.e.*, integrin α_M_β_2_-expressing) peripheral monocytes in an MHC class II independent mechanism [153]. This finding indicates that GA may form stable ligation to receptors on monocyte cell surface over a significant time span. Monocytes are usually considered a part of the innate immune system, but their potential to differentiate into macrophages and possibly myeloid-derived dendritic cells also enables functions in the acquired immune system, most notably with regard to regulating T cell functions. This is important to note since the many reported influences on the adaptive immunity by GA treatment mentioned above at the very least could come about through an indirect route targeting myeloid cells of the innate immune system. Any interaction between integrin α_M_β_2_ and MBP, or fragments hereof, in blood is purely speculative. In principle, the MBP found in the cerebrospinal fluid [141] could leak through a damaged BBB into plasma as it has been reported for both acute brain damage as well as chronic progressive neurological disorders [154]. However, yet other roles of the influence of GA on the function of α_M_β_2_ may be suggested. In a clinical setting, Sellebjerg *et al.* reported that GA treatment increased the number of circulating monocytes with approximately 20% [84]. Obviously, these effects of GA treatment are taking place outside the CNS. While the mechanistic explanation for this finding remains to be explored, one possibility is that GA releases the otherwise adherent monocytes through GA’s ligand binding-antagonistic properties towards the integrin α_M_β_2_. The GA-activity on monocyte function is of considerable interest since recent findings have indicated this leukocyte subset to play a major role in processes affected by MS [149,150] apparently through the ability of monocytes to modulate the function of T lymphocytes. The findings by Toker *et al.*[153] are strong evidence that the GA co-polymers may target integrin α_M_β_2_ under physiological conditions affecting this leukocyte subset of the innate immune system.

Alternative modes of action of GA may include a neuro-protective activity [129], apparently in a way connected to the ability of the drug to skew the inflammation towards a Th2 type humoral immune response. In a mouse model, accumulated GA-specific cells have been reported to express brain-derived neurotrophic factor (BDNF) *in situ* as detected by BDNF-immunostaining and confocal microscopy [155]. Interestingly, BDNF production may also be induced in dendritic cells following Cop-1 treatment. With regard to the humoral response, GA-reactive antibodies do not reduce the clinical efficiency of GA [83]. Indeed, Ure *et al.* showed that GA-reactive antibodies mediate positive effect on re-myelination [156]. This effect is not limited to EAE and can be observed in other models of encephalitis, one example being neuroprotecting effects in a murine model HIV-induced encephalitis.

## 6. Conclusions

As yet, no curative treatment exists for MS. However, it is clear that biological therapy targeting functions of the immune system presents several benefits, although strong suppression of the immune system is associated with severe opportunistic infections as a side effect. Particularly from this perspective, the efficacy of GA treatment is of interest. This review of the current knowledge on the PMA of GA, points to several routes targeting the immune system. Remarkably, in addition to the effects on the adaptive immune system, more recent findings now suggest that cellular components of the innate immune system are targeted, at least partially, through cell adhesion molecules such as integrin α_M_β_2_. From a biochemical point of view, it is perhaps not surprising that several PMA are at play when treating patients with random co-polymers of such diversity as in the case of GA. A more detailed understanding of these PMA is likely to support the development of a more potent drug. Furthermore, an important aspect is the development of new types of random copolymers for anti-inflammatory treatment. The case of GA suggests that such strategies may hold particular promise when several inflammatory responses are part of the pathogenic processes, which is likely to expand the use of GA or similar drugs to aid additional inflammatory disorders.

## References

[1] Charcot J. (1868). Histologie de la sclerose en plaques. Gaz. Hop.

[2] Kachuck N.J. (2009). Sustained release oral fampridine in the treatment of multiple sclerosis. Expert Opin. Pharmacother.

[3] Compston A., Coles A. (2008). Multiple sclerosis. Lancet.

[4] Sadovnick A.D., Ebers G.C. (1993). Epidemiology of multiple sclerosis: A critical overview. Can. J. Neurol. Sci.

[5] Calabresi P.A. (2004). Diagnosis and management of multiple sclerosis. Am. Family Phys.

[6] Rosati G. (2001). The prevalence of multiple sclerosis in the world: An update. Neurol. Sci.: Off. J. Ital. Neurol. Soc. Ital. Soc. Clin. Neurophysiol.

[7] Stys P.K., Zamponi G.W., van Minnen J., Geurts J.J. (2012). Will the real multiple sclerosis please stand up?. Nat. Rev. Neurosci.

[8] Compston A. (1997). Genetic epidemiology of multiple sclerosis. J. Neurol. Neurosurg. Psychiatry.

[9] Ota K., Matsui M., Milford E.L., Mackin G.A., Weiner H.L., Hafler D.A. (1990). T-cell recognition of an immunodominant myelin basic protein epitope in multiple sclerosis. Nature.

[10] Frohman E.M., Racke M.K., Raine C.S. (2006). Multiple sclerosis—the plaque and its pathogenesis. N. Engl. J. Med.

[11] Ascherio A., Munger K.L. (2007). Environmental risk factors for multiple sclerosis. Part I: The role of infection. Ann. Neurol.

[12] Ascherio A., Munger K.L. (2007). Environmental risk factors for multiple sclerosis. Part II: Noninfectious factors. Ann. Neurol.

[13] Ebers G.C. (2008). Environmental factors and multiple sclerosis. Lancet Neurol.

[14] Palacios N., Alonso A., Bronnum-Hansen H., Ascherio A. (2011). Smoking and increased risk of multiple sclerosis: Parallel trends in the sex ratio reinforce the evidence. Ann. Epidemiol.

[15] Mohr D.C., Hart S.L., Julian L., Cox D., Pelletier D. (2004). Association between stressful life events and exacerbation in multiple sclerosis: A meta-analysis. BMJ.

[16] Harkiolaki M., Holmes S.L., Svendsen P., Gregersen J.W., Jensen L.T., McMahon R., Friese M.A., van Boxel G., Etzensperger R., Tzartos J.S. (2009). T cell-mediated autoimmune disease due to low-affinity crossreactivity to common microbial peptides. Immunity.

[17] Levin L.I., Munger K.L., Rubertone M.V., Peck C.A., Lennette E.T., Spiegelman D., Ascherio A. (2003). Multiple sclerosis and Epstein-Barr virus. JAMA.

[18] DeLorenze G.N., Munger K.L., Lennette E.T., Orentreich N., Vogelman J.H., Ascherio A. (2006). Epstein-Barr virus and multiple sclerosis: Evidence of association from a prospective study with long-term follow-up. Arch. Neurol.

[19] Haahr S., Hollsberg P. (2006). Multiple sclerosis is linked to Epstein-Barr virus infection. Rev. Med. Virol.

[20] Christensen T. (2010). HERVs in neuropathogenesis. J. Neuroimmune Pharmacol.

[21] Sharma P., Azebi S., England P., Christensen T., Moller-Larsen A., Petersen T., Batsche E., Muchardt C. (2012). Citrullination of Histone H3 Interferes with HP1-Mediated Transcriptional Repression. PLoS Genet.

[22] Harauz G., Ishiyama N., Hill C.M., Bates I.R., Libich D.S., Fares C. (2004). Myelin basic protein-diverse conformational states of an intrinsically unstructured protein and its roles in myelin assembly and multiple sclerosis. Micron.

[23] Harauz G., Musse A.A. (2007). A tale of two citrullines—structural and functional aspects of myelin basic protein deimination in health and disease. Neurochem. Res.

[24] Musse A.A., Harauz G. (2007). Molecular “negativity” may underlie multiple sclerosis: Role of the myelin basic protein family in the pathogenesis of MS. Int. Rev. Neurobiol.

[25] Carrillo-Vico A., Leech M.D., Anderton S.M. (2010). Contribution of myelin autoantigen citrullination to T cell autoaggression in the central nervous system. J. Immunol.

[26] Sawcer S., Hellenthal G., Pirinen M., Spencer C.C., Patsopoulos N.A., Moutsianas L., Dilthey A., Su Z., Freeman C., Hunt S.E. (2011). Genetic risk and a primary role for cell-mediated immune mechanisms in multiple sclerosis. Nature.

[27] Jersild C., Svejgaard A., Fog T. (1972). HL-A antigens and multiple sclerosis. Lancet.

[28] Naito S., Namerow N., Mickey M.R., Terasaki P.I. (1972). Multiple sclerosis: Association with HL-A3. Tissue Antigen.

[29] Ramagopalan S.V., Maugeri N.J., Handunnetthi L., Lincoln M.R., Orton S.M., Dyment D.A., Deluca G.C., Herrera B.M., Chao M.J., Sadovnick A.D. (2009). Expression of the multiple sclerosis-associated MHC class II Allele HLA-DRB1*1501 is regulated by vitamin D. PLoS Genet.

[30] Friese M.A., Jakobsen K.B., Friis L., Etzensperger R., Craner M.J., McMahon R.M., Jensen L.T., Huygelen V., Jones E.Y., Bell J.I. (2008). Opposing effects of HLA class I molecules in tuning autoreactive CD8+ T cells in multiple sclerosis. Nat. Med.

[31] Madsen L.S., Andersson E.C., Jansson L., krogsgaard M., Andersen C.B., Engberg J., Strominger J.L., Svejgaard A., Hjorth J.P., Holmdahl R. (1999). A humanized model for multiple sclerosis using HLA-DR2 and a human T-cell receptor. Nat. Genet.

[32] Lublin F.D., Reingold S.C. (1996). Defining the clinical course of multiple sclerosis: Results of an international survey. National Multiple Sclerosis Society (USA) Advisory Committee on Clinical Trials of New Agents in Multiple Sclerosis. Neurology.

[33] Miller D., Barkhof F., Montalban X., Thompson A., Filippi M. (2005). Clinically isolated syndromes suggestive of multiple sclerosis, part 2: Non-conventional MRI, recovery processes, and management. Lancet Neurol.

[34] Miller D.H. (2004). Biomarkers and surrogate outcomes in neurodegenerative disease: Lessons from multiple sclerosis. NeuroRx.

[35] Flachenecker P., Hartung H.P. (1996). Course of illness and prognosis of multiple sclerosis. 1: The natural illness course. Nervenarzt.

[36] Moura A.L., Teixeira R.A., Oiwa N.N., Costa M.F., Feitosa-Santana C., Callegaro D., Hamer R.D., Ventura D.F. (2008). Chromatic discrimination losses in multiple sclerosis patients with and without optic neuritis using the Cambridge Colour Test. Vis. Neurosci.

[37] Patterson V.H., Heron J.R. (1980). Visual field abnormalities in multiple sclerosis. J. Neurol. Neurosurg. Psychiatry.

[38] Leray E., Yaouanq J., le Page E., Coustans M., Laplaud D., Oger J., Edan G. (2010). Evidence for a two-stage disability progression in multiple sclerosis. Brain.

[39] Bronnum-Hansen H., Koch-Henriksen N., Stenager E. (2004). Trends in survival and cause of death in Danish patients with multiple sclerosis. Brain.

[40] Weinshenker B.G. (1994). Natural history of multiple sclerosis. Ann. Neurol.

[41] Beiske A.G., Pedersen E.D., Czujko B., Myhr K.M. (2004). Pain and sensory complaints in multiple sclerosis. Eur. J. Neurol.

[42] Feinstein A. (2011). Multiple sclerosis and depression. Mult. Scler.

[43] Andersson M., Alvarez-Cermeno J., Bernardi G., Cogato I., Fredman P., Frederiksen J., Fredrikson S., Gallo P., Grimaldi L.M., Gronning M. (1994). Cerebrospinal fluid in the diagnosis of multiple sclerosis: A consensus report. J. Neurol. Neurosurg. Psychiatry.

[44] Polman C.H., Reingold S.C., Edan G., Filippi M., Hartung H.P., Kappos L., Lublin F.D., Metz L.M., McFarland H.F., O’Connor P.W. (2005). Diagnostic criteria for multiple sclerosis: 2005 revisions to the “McDonald Criteria”. Ann. Neurol.

[45] McDonald W.I., Compston A., Edan G., Goodkin D., Hartung H.P., Lublin F.D., McFarland H.F., Paty D.W., Polman C.H., Reingold S.C. (2001). Recommended diagnostic criteria for multiple sclerosis: Guidelines from the International Panel on the diagnosis of multiple sclerosis. Ann. Neurol.

[46] Whiting P., Harbord R., Main C., Deeks J.J., Filippini G., Egger M., Sterne J.A. (2006). Accuracy of magnetic resonance imaging for the diagnosis of multiple sclerosis: systematic review. BMJ.

[47] Miller D.M., Weinstock-Guttman B., Bethoux F., Lee J.C., Beck G., Block V., Durelli L., LaMantia L., Barnes D., Sellebjerg F. (2000). A meta-analysis of methylprednisolone in recovery from multiple sclerosis exacerbations. Mult. Scler.

[48] Martinelli Boneschi F., Rovaris M., Capra R., Comi G. (2005). Mitoxantrone for multiple sclerosis. Cochrane Database Syst. Rev..

[49] Swinburn W.R., Liversedge L.A. (1973). Long-term treatment of multiple sclerosis with azathioprine. J. Neurol. Neurosurg. Psychiatry.

[50] Casetta I., Iuliano G., Filippini G. (2009). Azathioprine for multiple sclerosis. J. Neurol. Neurosurg. Psychiatry.

[51] Lugaresi A., Caporale C., Farina D., Marzoli F., Bonanni L., Muraro P.A., de Luca G., Iarlori C., Gambi D. (2001). Low-dose oral methotrexate treatment in chronic progressive multiple sclerosis. Neurol. Sci.

[52] Goodkin D.E., Rudick R.A., VanderBrug Medendorp S., Greene T., Schwetz K.M., Fischer J., Daughtry M.M., Ross J., Van Dyke C. (1992). Low-dose (7.5 mg) oral methotrexate for chronic progressive multiple sclerosis. Design of a randomized, placebo-controlled trial with sample size benefits from a composite outcome variable including preliminary data on toxicity. Online J. Curr. Clin. Trials.

[53] Weitz-Schmidt G., Welzenbach K., Brinkmann V., Kamata T., Kallen J., Bruns C., Cottens S., Takada Y., Hommel U. (2001). Statins selectively inhibit leukocyte function antigen-1 by binding to a novel regulatory integrin site. Nat. Med.

[54] Kallen J., Welzenbach K., Ramage P., Geyl D., Kriwacki R., Legge G., Cottens S., Weitz-Schmidt G., Hommel U. (1999). Structural basis for LFA-1 inhibition upon lovastatin binding to the CD11a I-domain. J. Mol. Biol.

[55] Katznelson S., Kobashigawa J.A. (1995). Dual roles of HMG-CoA reductase inhibitors in solid organ transplantation: lipid lowering and immunosuppression. Kidney Int. Suppl.

[56] Wang J., Xiao Y., Luo M., Luo H. (2011). Statins for multiple sclerosis. Cochrane Database Syst. Rev..

[57] Cortese I., Chaudhry V., So Y.T., Cantor F., Cornblath D.R., Rae-Grant A. (2011). Evidence-based guideline update: Plasmapheresis in neurologic disorders: report of the Therapeutics and Technology Assessment Subcommittee of the American Academy of Neurology. Neurology.

[58] Achiron A., Kishner I., Sarova-Pinhas I., Raz H., Faibel M., Stern Y., Lavie M., Gurevich M., Dolev M., Magalashvili D. (2004). Intravenous immunoglobulin treatment following the first demyelinating event suggestive of multiple sclerosis: A randomized, double-blind, placebo-controlled trial. Arch. Neurol.

[59] Bohn A.B., Nederby L., Harbo T., Skovbo A., Vorup-Jensen T., Krog J., Jakobsen J., Hokland M.E. (2011). The effect of IgG levels on the number of natural killer cells and their Fc receptors in chronic inflammatory demyelinating polyradiculoneuropathy. Eur. J. Neurol.

[60] Kappos L., European Study Group on interferon beta-1b in secondary progressive MS (1998). Placebo-controlled multicentre randomised trial of interferon beta-1b in treatment of secondary progressive multiple sclerosis. Lancet.

[61] Jacobs L.D., Beck R.W., Simon J.H., Kinkel R.P., Brownscheidle C.M., Murray T.J., Simonian N.A., Slasor P.J., Sandrock A.W., CHAMPS Study Group (2000). Intramuscular interferon beta-1a therapy initiated during a first demyelinating event in multiple sclerosis. N. Engl. J. Med..

[62] Comi G., Filippi M., Barkhof F., Durelli L., Edan G., Fernandez O., Hartung H., Seeldrayers P., Sorensen P.S., Rovaris M. (2001). Effect of early interferon treatment on conversion to definite multiple sclerosis: A randomised study. Lancet.

[63] Yednock T.A., Cannon C., Fritz L.C., Sanchez-Madrid F., Steinman L., Karin N. (1992). Prevention of experimental autoimmune encephalomyelitis by antibodies against alpha 4 beta 1 integrin. Nature.

[64] Steinman L., Zamvil S.S. (2005). Virtues and pitfalls of EAE for the development of therapies for multiple sclerosis. Trends Immunol.

[65] Polman C.H., O’Connor P.W., Havrdova E., Hutchinson M., Kappos L., Miller D.H., Phillips J.T., Lublin F.D., Giovannoni G., Wajgt A. (2006). A randomized, placebo-controlled trial of natalizumab for relapsing multiple sclerosis. N. Engl. J. Med.

[66] Miller D.H., Soon D., Fernando K.T., MacManus D.G., Barker G.J., Yousry T.A., Fisher E., O’Connor P.W., Phillips J.T., Polman C.H. (2007). MRI outcomes in a placebo-controlled trial of natalizumab in relapsing MS. Neurology.

[67] Sorensen P.S., Ross C., Clemmesen K.M., Bendtzen K., Frederiksen J.L., Jensen K., Kristensen O., Petersen T., Rasmussen S., Ravnborg M. (2003). Clinical importance of neutralising antibodies against interferon beta in patients with relapsing-remitting multiple sclerosis. Lancet.

[68] Calabresi P.A., Giovannoni G., Confavreux C., Galetta S.L., Havrdova E., Hutchinson M., Kappos L., Miller D.H., O’Connor P.W., Phillips J.T. (2007). The incidence and significance of anti-natalizumab antibodies: Results from AFFIRM and SENTINEL. Neurology.

[69] Bloomgren G., Richman S., Hotermans C., Subramanyam M., Goelz S., Natarajan A., Lee S., Plavina T., Scanlon J.V., Sandrock A. (2012). Risk of natalizumab-associated progressive multifocal leukoencephalopathy. N. Engl. J. Med.

[70] Tavazzi E., Ferrante P., Khalili K. (2011). Progressive multifocal leukoencephalopathy: An unexpected complication of modern therapeutic monoclonal antibody therapies. Clin. Microbiol. Infect.

[71] Vorup-Jensen T. (2012). On the roles of polyvalent binding in immune recognition: Perspectives in the nanoscience of immunology and the immune response to nanomedicines. Adv. Drug Deliv. Rev..

[72] Krumbholz M., Derfuss T., Hohlfeld R., Meinl E. (2012). B cells and antibodies in multiple sclerosis pathogenesis and therapy. Nat. Rev. Neurol..

[73] Bornstein M.B., Miller A., Slagle S., Weitzman M., Crystal H., Drexler E., Keilson M., Merriam A., Wassertheil-Smoller S., Spada V. (1987). A pilot trial of Cop 1 in exacerbating-remitting multiple sclerosis. N. Engl. J. Med.

[74] Miller A., Spada V., Beerkircher D., Kreitman R.R. (2008). Long-term (up to 22 years), open-label, compassionate-use study of glatiramer acetate in relapsing-remitting multiple sclerosis. Mult. Scler.

[75] Ford C., Goodman A.D., Johnson K., Kachuck N., Lindsey J.W., Lisak R., Luzzio C., Myers L., Panitch H., Preiningerova J. (2010). Continuous long-term immunomodulatory therapy in relapsing multiple sclerosis: Results from the 15-year analysis of the US prospective open-label study of glatiramer acetate. Mult. Scler.

[76] Ford C.C., Johnson K.P., Lisak R.P., Panitch H.S., Shifronis G., Wolinsky J.S. (2006). A prospective open-label study of glatiramer acetate: Over a decade of continuous use in multiple sclerosis patients. Mult. Scler.

[77] Comi G., Filippi M., Wolinsky J.S. (2001). European/Canadian multicenter, double-blind, randomized, placebo-controlled study of the effects of glatiramer acetate on magnetic resonance imaging—measured disease activity and burden in patients with relapsing multiple sclerosis. European/Canadian Glatiramer Acetate Study Group. Ann. Neurol.

[78] Johnson K.P., Brooks B.R., Cohen J.A., Ford C.C., Goldstein J., Lisak R.P., Myers L.W., Panitch H.S., Rose J.W., Schiffer R.B., The Copolymer 1 Multiple Sclerosis Study Group (1995). Copolymer 1 reduces relapse rate and improves disability in relapsing-remitting multiple sclerosis: Results of a phase III multicenter, double-blind placebo-controlled trial. Neurology.

[79] Martinelli Boneschi F., Rovaris M., Johnson K.P., Miller A., Wolinsky J.S., Ladkani D., Shifroni G., Comi G., Filippi M. (2003). Effects of glatiramer acetate on relapse rate and accumulated disability in multiple sclerosis: Meta-analysis of three double-blind, randomized, placebo-controlled clinical trials. Mult. Scler.

[80] La Mantia L., Munari L.M., Lovati R. (2010). Glatiramer acetate for multiple sclerosis. Cochrane Database Syst. Rev..

[81] Korczyn A.D., Nisipeanu P. (1996). Safety profile of copolymer 1: Analysis of cumulative experience in the United States and Israel. J. Neurol.

[82] Copaxone 20mg/mL, Solution For Injection, Pre-Filled Syringe.

[83] Johnson K.P., Brooks B.R., Ford C.C., Goodman A., Guarnaccia J., Lisak R.P., Myers L.W., Panitch H.S., Pruitt A., Rose J.W. (2000). Sustained clinical benefits of glatiramer acetate in relapsing multiple sclerosis patients observed for 6 years. Copolymer 1 Multiple Sclerosis Study Group. Mult. Scler.

[84] Sellebjerg F., Hedegaard C.J., Krakauer M., Hesse D., Lund H., Nielsen C.H., Sondergaard H.B., Sorensen P.S. (2012). Glatiramer acetate antibodies, gene expression and disease activity in multiple sclerosis. Mult. Scler.

[85] Sela M. (1998). Poly(α-amino acids)—From a better understanding of immune phenomena to a drug against multiple sclerosis. Acta Polym.

[86] Arnon R., Sela M. (2003). Immunomodulation by the copolymer glatiramer acetate. J. Mol. Recognit.

[87] Arnon R., Sela M. (1999). The chemistry of the Copaxone drug. Chem. Isr.

[88] Katchalski-Katzir E. (2005). My contributions to science and society. J. Biol. Chem.

[89] Eisenbach M. (2009). Ephraim Katchalski-Katzir (1916–2009). Trends Biochem. Sci.

[90] Katchalski E., Grossfeld I., Frankel M. (1947). Poly-lysine. J. Am. Chem. Soc.

[91] Katchalski E., Sela M. (1958). Synthesis and chemical properties of poly-alpha-amino acids. Adv. Protein Chem.

[92] Pauling L., Corey R.B., Branson H.R. (1951). The structure of proteins; two hydrogen-bonded helical configurations of the polypeptide chain. Proc. Natl. Acad. Sci. USA.

[93] Perutz M.F. (1951). New X-ray evidence on the configuration of polypeptide chains. Nature.

[94] Rich A., Crick F.H. (1955). The structure of collagen. Nature.

[95] Engel J., Kurtz J., Katchalski E., Berger A. (1966). Polymers tripeptides as collagen models. II. Conformational changes of poly(l-prolyl-glycyl-l-prolyl) in solution. J. Mol. Biol.

[96] Levin Y., Berger A., Katchalski E. (1956). Hydrolysis and transpeptidation of lysine peptides by trypsin. Biochem. J.

[97] Arnon R., Sela M. (1960). Studies on the chemical basis of the antigenicity of proteins. 2. Antigenic specificity of polytyrosyl gelatins. Biochem. J.

[98] Sela M., Arnon R. (1960). Studies on the chemical basis of the antigenicity of proteins. 1. Antigenicity of polypeptidyl gelatins. Biochem. J.

[99] Sela M., Arnon R. (1960). Studies on the chemical basis of the antigenicity of proteins. 3. The role of rigidity in the antigenicity of polypeptidyl gelatins. Biochem. J.

[100] Arnon R., Maron E., Sela M., Anfinsen C.B. (1971). Antibodies reactive with native lysozyme elicited by a completely synthetic antigen. Proc. Natl. Acad. Sci. USA.

[101] Shaw C.M., Alvord E.C., Fahlberg W.J., Kies M.W. (1962). Specificity of encephalitogen-induced inhibition of experimental “allergic” encephalomyelitis in the guinea pig. J. Immunol..

[102] Teitelbaum D., Meshorer A., Hirshfeld T., Arnon R., Sela M. (1971). Suppression of experimental allergic encephalomyelitis by a synthetic polypeptide. Eur. J. Immunol.

[103] Teitelbaum D., Gan R., Meshorer A., Hirsfeld T., Arnon R., Sela M. (1971). Therapeutic Copolymer. U.S. Patent.

[104] Katchalski-Katzir E. (1996). Synthesis, physicochemical and biological properties of poly-alpha-amino acids--the simplest of protein models. Acta Biochim. Polon.

[105] Fridkis-Hareli M., Santambrogio L., Stern J.N., Fugger L., Brosnan C., Strominger J.L. (2002). Novel synthetic amino acid copolymers that inhibit autoantigen-specific T cell responses and suppress experimental autoimmune encephalomyelitis. J. Clin. Invest.

[106] Capila I., Linhardt R.J. (2002). Heparin-protein interactions. Angew. Chem. Int. Ed. Engl.

[107] Venkataraman G., Shriver Z., Raman R., Sasisekharan R. (1999). Sequencing complex polysaccharides. Science.

[108] Berkowitz S.A., Engen J.R., Mazzeo J.R., Jones G.B. (2012). Analytical tools for characterizing biopharmaceuticals and the implications for biosimilars. Nat. Rev. Drug Discov.

[109] Guerrini M., Beccati D., Shriver Z., Naggi A., Viswanathan K., Bisio A., Capila I., Lansing J.C., Guglieri S., Fraser B. (2008). Oversulfated chondroitin sulfate is a contaminant in heparin associated with adverse clinical events. Nat. Biotechnol.

[110] Kishimoto T.K., Viswanathan K., Ganguly T., Elankumaran S., Smith S., Pelzer K., Lansing J.C., Sriranganathan N., Zhao G., Galcheva-Gargova Z. (2008). Contaminated heparin associated with adverse clinical events and activation of the contact system. N. Engl. J. Med.

[111] Liu H., Zhang Z., Linhardt R.J. (2009). Lessons learned from the contamination of heparin. Nat. Prod. Rep.

[112] Fridkis-Hareli M., Stern J.N., Fugger L., Strominger J.L. (2001). Synthetic peptides that inhibit binding of the myelin basic protein 85–99 epitope to multiple sclerosis-associated HLA-DR2 molecules and MBP-specific T-cell responses. Human Immunol.

[113] Teitelbaum D., Aharoni R., Sela M., Arnon R. (1991). Cross-reactions and specificities of monoclonal antibodies against myelin basic protein and against the synthetic copolymer 1. Proc. Natl. Acad. Sci. USA.

[114] Fridkis-Hareli M., Strominger J.L. (1998). Promiscuous binding of synthetic copolymer 1 to purified HLA-DR molecules. J. Immunol.

[115] Fridkis-Hareli M., Aharoni R., Teitelbaum D., Arnon R., Sela M., Strominger J.L. (1999). Binding of random copolymers of three amino acids to class II MHC molecules. Int. Immunol.

[116] Teitelbaum D., Fridkis-Hareli M., Arnon R., Sela M. (1996). Copolymer 1 inhibits chronic relapsing experimental allergic encephalomyelitis induced by proteolipid protein (PLP) peptides in mice and interferes with PLP-specific T cell responses. J. Neuroimmunol.

[117] Ben-Nun A., Mendel I., Bakimer R., Fridkis-Hareli M., Teitelbaum D., Arnon R., Sela M., de Rosbo N.K. (1996). The autoimmune reactivity to myelin oligodendrocyte glycoprotein (MOG) in multiple sclerosis is potentially pathogenic: effect of copolymer 1 on MOG-induced disease. J. Neurol.

[118] Ibarra A., Avendano H., Cruz Y. (2007). Copolymer-1 (Cop-1) improves neurological recovery after middle cerebral artery occlusion in rats. Neurosci. Lett.

[119] Aharoni R., Teitelbaum D., Arnon R., Sela M. (2001). Copolymer 1 inhibits manifestations of graft rejection. Transplantation.

[120] Duda P.W., Schmied M.C., Cook S.L., Krieger J.I., Hafler D.A. (2000). Glatiramer acetate (Copaxone) induces degenerate, Th2-polarized immune responses in patients with multiple sclerosis. J. Clin. Invest.

[121] Berthelot L., Miqueu P., Pettre S., Guillet M., Moynard J., Wiertlewski S., Lefrere F., Brouard S., Soulillou J.P., Laplaud D.A. (2010). Failure of glatiramer acetate to modify the peripheral T cell repertoire of relapsing-remitting multiple sclerosis patients. Clin. Immunol.

[122] Aharoni R., Teitelbaum D., Sela M., Arnon R. (1997). Copolymer 1 induces T cells of the T helper type 2 that crossreact with myelin basic protein and suppress experimental autoimmune encephalomyelitis. Proc. Natl. Acad. Sci. USA.

[123] Kala M., Rhodes S.N., Piao W.H., Shi F.D., Campagnolo D.I., Vollmer T.L. (2010). B cells from glatiramer acetate-treated mice suppress experimental autoimmune encephalomyelitis. Exp. Neurol.

[124] Racke M.K., Lovett-Racke A.E. (2011). Glatiramer acetate treatment of multiple sclerosis: an immunological perspective. J. Immunol.

[125] Neuhaus O., Farina C., Yassouridis A., Wiendl H., Then Bergh F., Dose T., Wekerle H., Hohlfeld R. (2000). Multiple sclerosis: Comparison of copolymer-1- reactive T cell lines from treated and untreated subjects reveals cytokine shift from T helper 1 to T helper 2 cells. Proc. Natl. Acad. Sci. USA.

[126] Krogsgaard M., Wucherpfennig K.W., Cannella B., Hansen B.E., Svejgaard A., Pyrdol J., Ditzel H., Raine C., Engberg J., Fugger L. (2000). Visualization of myelin basic protein (MBP) T cell epitopes in multiple sclerosis lesions using a monoclonal antibody specific for the human histocompatibility leukocyte antigen (HLA)-DR2-MBP 85–99 complex. J. Exp. Med.

[127] Aharoni R., Teitelbaum D., Sela M., Arnon R. (1998). Bystander suppression of experimental autoimmune encephalomyelitis by T cell lines and clones of the Th2 type induced by copolymer 1. J. Neuroimmunol.

[128] Jee Y., Liu R., Bai X.F., Campagnolo D.I., Shi F.D., Vollmer T.L. (2006). Do Th2 cells mediate the effects of glatiramer acetate in experimental autoimmune encephalomyelitis?. Int. Immunol.

[129] Kipnis J., Yoles E., Porat Z., Cohen A., Mor F., Sela M., Cohen I.R., Schwartz M. (2000). T cell immunity to copolymer 1 confers neuroprotection on the damaged optic nerve: possible therapy for optic neuropathies. Proc. Natl. Acad. Sci. USA.

[130] Stapulionis R., Oliveira C.L.P., Gjelstrup M.C., Pedersen J.S., Hokland M.E., Hoffmann S.V., Poulsen K., Jacobsen C., Vorup-Jensen T. (2008). Structural insight into the function of myelin basic protein as a ligand for integrin alpha(M)beta(2). J. Immunol.

[131] Rathinam V.A., Vanaja S.K., Fitzgerald K.A. (2012). Regulation of inflammasome signaling. Nat. Immunol..

[132] Gordon S. (2002). Pattern recognition receptors: doubling up for the innate immune response. Cell.

[133] Hynes R.O. (2002). Integrins: bidirectional, allosteric signaling machines. Cell.

[134] Springer T.A. (1990). Adhesion receptors of the immune system. Nature.

[135] Luo B.H., Carman C.V., Springer T.A. (2007). Structural basis of integrin regulation and signaling. Annu. Rev. Immunol.

[136] Gomez I.G., Tang J., Wilson C.L., Yan W., Heinecke J.W., Harlan J.M., Raines E.W. (2012). Metalloproteinase-mediated Shedding of Integrin beta2 promotes macrophage efflux from inflammatory sites. J. Biol. Chem.

[137] Gjelstrup L.C., Boesen T., Kragstrup T.W., Jorgensen A., Klein N.J., Thiel S., Deleuran B.W., Vorup-Jensen T. (2010). Shedding of large functionally active CD11/CD18 Integrin complexes from leukocyte membranes during synovial inflammation distinguishes three types of arthritis through differential epitope exposure. J. Immunol.

[138] Vorup-Jensen T., Carman C.V., Shimaoka M., Schuck P., Svitel J., Springer T.A. (2005). Exposure of acidic residues as a danger signal for recognition of fibrinogen and other macromolecules by integrin alphaXbeta2. Proc. Natl. Acad. Sci. USA.

[139] Vorup-Jensen T., Chi L., Gjelstrup L.C., Jensen U.B., Jewett C.A., Xie C., Shimaoka M., Linhardt R.J., Springer T.A. (2007). Binding between the integrin alphaXbeta2 (CD11c/CD18) and heparin. J. Biol. Chem.

[140] Davis G.E. (1992). The Mac-1 and p150,95 beta 2 integrins bind denatured proteins to mediate leukocyte cell-substrate adhesion. Exp. Cell. Res.

[141] Whitaker J.N. (1998). Myelin basic protein in cerebrospinal fluid and other body fluids. Mult. Scler.

[142] Constantinescu R., Zetterberg H., Holmberg B., Rosengren L. (2009). Levels of brain related proteins in cerebrospinal fluid: an aid in the differential diagnosis of parkinsonian disorders. Parkinsonism Relat. Disord.

[143] Prineas J.W., Graham J.S. (1981). Multiple sclerosis: Capping of surface immunoglobulin G on macrophages engaged in myelin breakdown. Ann. Neurol.

[144] Epstein L.G., Prineas J.W., Raine C.S. (1983). Attachment of myelin to coated pits on macrophages in experimental allergic encephalomyelitis. J. Neurol. Sci.

[145] Nielsen H.H., Ladeby R., Fenger C., Toft-Hansen H., Babcock A.A., Owens T., Finsen B. (2009). Enhanced microglial clearance of myelin debris in T cell-infiltrated central nervous system. J. Neuropathol. Exp. Neurol.

[146] Ransohoff R.M., Kivisakk P., Kidd G. (2003). Three or more routes for leukocyte migration into the central nervous system. Nat. Rev. Immunol.

[147] Lobel E., Riven-Kreitman R., Amselem A., Pinchasi I. (1996). Copolymer 1. Drug Fut.

[148] Ziemssen T., Neuhaus O., Hohlfeld R. (2001). Risk-benefit assessment of glatiramer acetate in multiple sclerosis. Drug Saf.

[149] Weber M.S., Prod’homme T., Youssef S., Dunn S.E., Rundle C.D., Lee L., Patarroyo J.C., Stuve O., Sobel R.A., Steinman L. (2007). Type II monocytes modulate T cell-mediated central nervous system autoimmune disease. Nat. Med.

[150] Weber M.S., Starck M., Wagenpfeil S., Meinl E., Hohlfeld R., Farina C. (2004). Multiple sclerosis: glatiramer acetate inhibits monocyte reactivity *in vitro* and *in vivo*. Brain.

[151] Clausen J., Matzke J., Gerhardt W. (1964). Agar-Gel Micro-Electrophoresis of Proteins in the Cerebrospinal Fluid Normal and Pathological Findings. Acta Neurol. Scand. Suppl.

[152] Borlongan C.V., Glover L.E., Sanberg P.R., Hess D.C. (2012). Permeating the blood brain barrier and abrogating the inflammation in stroke: Implications for stroke therapy. Curr. Pharm. Des.

[153] Toker A., Slaney C.Y., Backstrom B.T., Harper J.L. (2011). Glatiramer acetate treatment directly targets CD11b(+)Ly6G(-) monocytes and enhances the suppression of autoreactive T cells in experimental autoimmune encephalomyelitis. Scand. J. Immunol.

[154] Lamers K.J., Vos P., Verbeek M.M., Rosmalen F., van Geel W.J., van Engelen B.G. (2003). Protein S-100B, neuron-specific enolase (NSE), myelin basic protein (MBP) and glial fibrillary acidic protein (GFAP) in cerebrospinal fluid (CSF) and blood of neurological patients. Brain Res. Bull.

[155] Aharoni R., Kayhan B., Eilam R., Sela M., Arnon R. (2003). Glatiramer acetate-specific T cells in the brain express T helper 2/3 cytokines and brain-derived neurotrophic factor *in situ*. Proc. Natl. Acad. Sci. USA.

[156] Ure D.R., Rodriguez M. (2002). Polyreactive antibodies to glatiramer acetate promote myelin repair in murine model of demyelinating disease. FASEB J.

